# Localization of small peripheral pulmonary nodules for surgical resection: a new intraoperative technique in hybrid operating room

**DOI:** 10.1186/s13019-022-02012-4

**Published:** 2022-09-29

**Authors:** Hanbo Yu, Wenxin Tian, Yaoguang Sun, Qingjun Wu, Chao Ma, Peng Jiao, Chuan Huang, Donghang Li, Hongfeng Tong

**Affiliations:** Department of Thoracic Surgery, Beijing Hospital, National Center of Gerontology, Institute of Geriatric Medicine, Chinese Academy of Medical Sciences, No. 1 Dahua Road, Dong Dan, Beijing, 100730 People’s Republic of China

**Keywords:** Localization, Pulmonary nodule, Intraoperative

## Abstract

**Objective:**

The purpose of this study was to introduce a new feasible and effective intraoperative localization technique for small peripheral pulmonary nodules in hybrid operating room.

**Methods:**

Between June 2020 and June 2021, the intraoperative localization was performed in 27 patients for 35 small pulmonary nodules at our institution. The procedure was undergone under thoracoscopic observation. After making the VATS ports, a titanium clip was clipped at the visceral pleura as near the pulmonary nodule as possible to be a marker for the nodule. VATS resection was performed next.

**Results:**

A total of 27 patients were included in this study, including 6 males and 21 females. The median age was 58 years (range 34–78 years). All surgeries were performed by two-port VATS. A total of 35 pulmonary nodules underwent intraoperative localization. The mean diameter of nodules was 10.6 ± 3.7 mm. The distance of nodules to visceral pleura was 8.3 ± 8.7 mm. The mean localization time was 23.3 ± 3.3 min. The median time of C-arm scanning was 3 (range 2–4) times. The median times for clipping were 2 (range 1–3) times. All the nodules were localized successfully and resected precisely. No VATS were converted to thoracotomy. There were no complications related to localization procedures.

**Conclusions:**

This new intraoperative localization technique was feasible, safe and effective. And also the intraoperative procedure could avoid extra suffering for patients.

## Background

With the widespread use of low-dose computed tomography (LDCT), more and more small pulmonary nodules are being detected. Surgical resection is the main treatment for those malignant nodules, and video-assisted thoracoscopic surgery (VATS) has been widely used as a minimally invasive way. However, small pulmonary nodules, especially ground-glass nodules, are often invisible or impalpable. Localization of these small nodules is quite essential and also helpful for definitive resection. Many localization methods have been previously reported, of which computed tomography (CT) guided localization procedure is the most widely used method [1–4]. But most of those methods are implemented preoperatively, which make patients suffer more pains whether physically or mentally. We conducted a new method of localization for small pulmonary nodules using titanium clips during VATS in hybrid operating room. In this study, we introduced this novel technique, and evaluated the effectiveness and safety.

## Methods

### Patients

Between June 2020 and June 2021, the intraoperative localization was performed in 27 patients for 35 small pulmonary nodules at our institution. High-resolution computed tomography (HRCT) was taken for all patients. This study was approved by the Institutional Ethics Review Board of Beijing Hospital. An informed consent was obtained from all patients before surgery.

The inclusion criteria for intraoperative localization were as follows: (1) pure ground-glass lesions; (2) mixed ground-glass lesions with solid component ≤ 50%; (3) solid nodules ≤ 1 cm in diameter; (4) subpleural nodules that were ≥ 5 mm from the visceral pleura. The exclusion criteria were as follows: (1) nodules with obvious pleural indentation sign; (2) nodules located in the inner third of lung field.

### Procedure for intraoperative localization and surgery

**Fig. 1:**
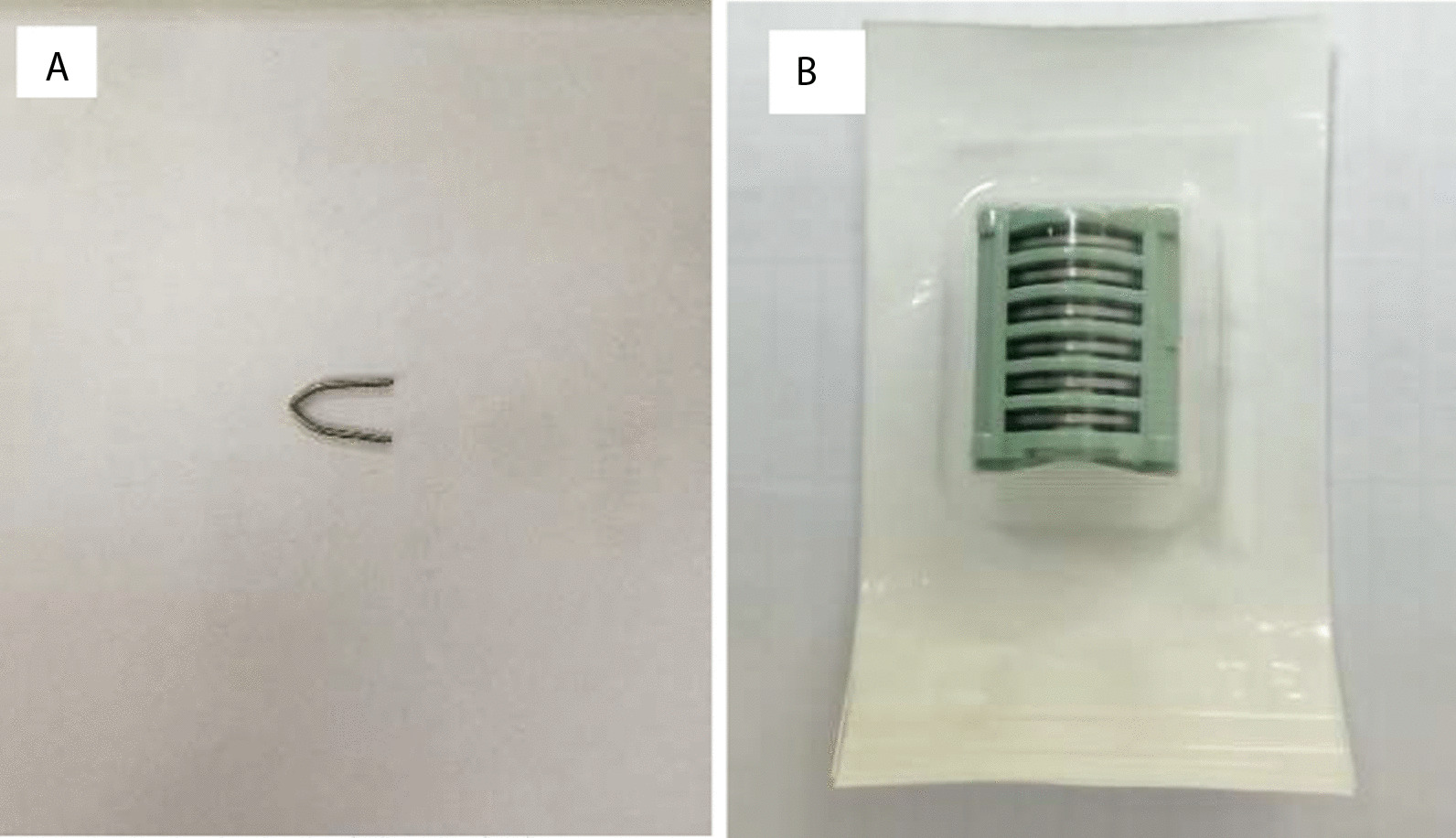
**A**, **B** Titanium clips (ETHICON Endo-Surgery 1 Ligaclip Extra Ligating Clip Cartridge 6 M Titanium Clips LT 300)

**Fig. 2:**
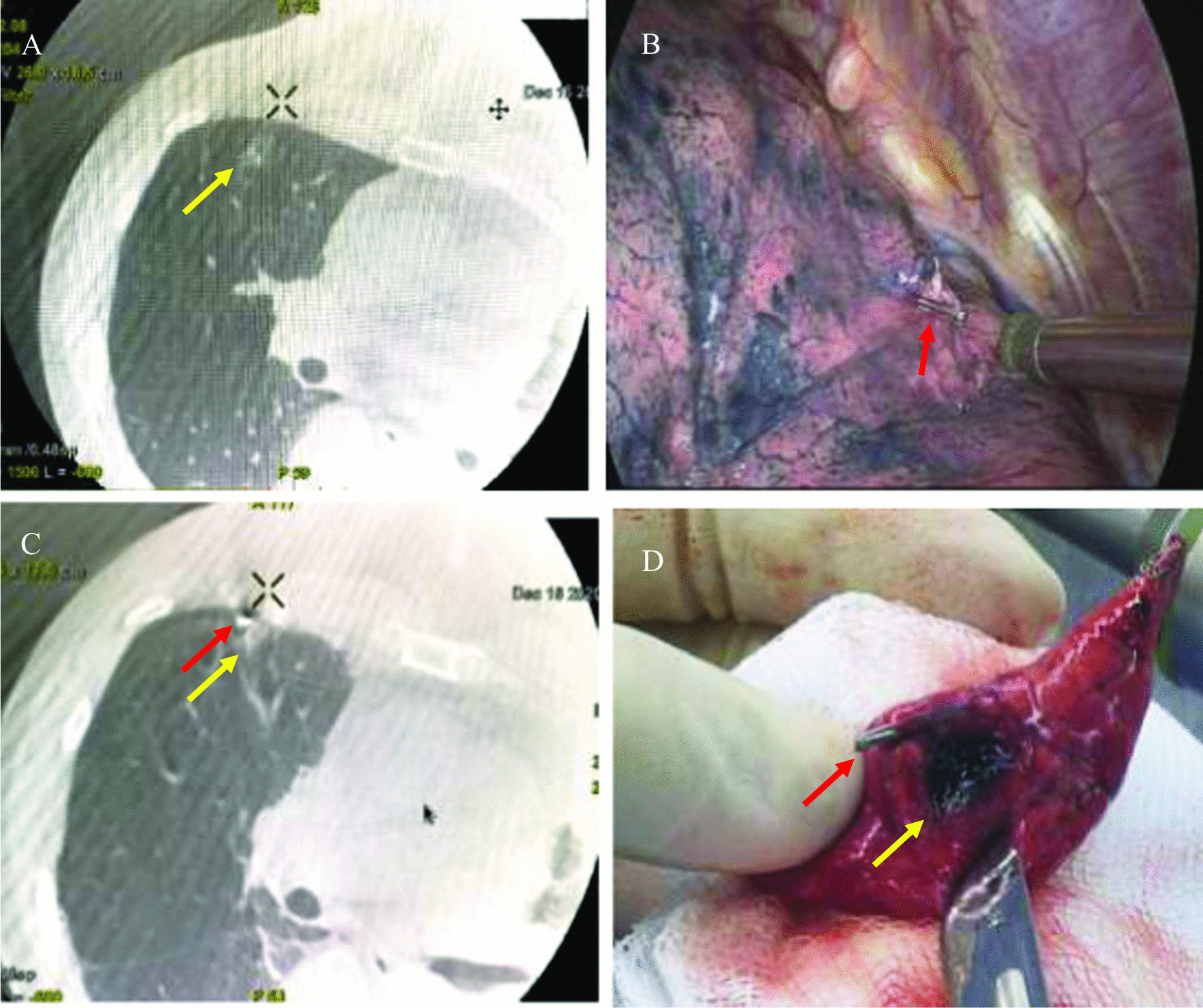
Imaging findings of intraoperative localization. **A** The baseline scanning image of pulmonary nodule located at right upper lobe. **B** A titanium clip was clipped at the visceral pleura as near the pulmonary nodule as possible under VATS observation. **C** Re-scanning image of the nodule after the titanium clip was clipped. **D** The pulmonary nodule was resected with endoscopic staplers using wedge resection. The red arrow showed the titanium clip. The yellow arrow showed the pulmonary nodule

All the operations were performed by two-port VATS in hybrid operating room with a C-arm X-ray machine (the Discovery IGS 7 angiography system, GE), which can bring extremely high-quality C-arm CT imaging. Under general anesthesia, a single-lumen tube with a blocker was used for intubation. Patients were placed in the healthy lateral decubitus position and the baseline scan was performed at the end of inhalation. Combined with preoperative HRCT, nodules needed to be localized were confirmed in the baseline scan imaging. After skin preparation and draping completed, incisions were made routinely. Usually, an observation port was made at the seventh or eighth intercostal space of the middle axillary line, and the utility port was made at the fourth or fifth intercostal space of the anterior axillary line. A titanium clip (Ethicon endo-surgery 1 ligaclip extra ligating clip cartridge 6 M titanium clips LT 300, Fig. 1) was clipped at the visceral pleura as near the pulmonary nodule as possible under VATS observation, according to anatomic landmarks showed in the baseline scan and preoperative CT imaging. The titanium clip was usually tightly clipped when the lung of the surgical side was inflated at a range of 80%-90%, in case of clipping position shifting or titanium clip dropping. Then the bilateral lung ventilation was restored and when the lung of the surgical side was inflated completely, scanning was performed again to confirm the relative position of the titanium clip and the nodule (Fig. 2). The ideal position of the titanium clip at the visceral pleura was the point which has the shortest straight-line distance to the nodule. If the titanium clip was far away from the ideal position, adjustment should be made. The horizontal and vertical distance were measured from the titanium clip to the ideal position. Then, under VATS observation, a new titanium was clipped, and the first titanium clip was removed. A third scanning was performed as previously described, to confirm the position of the new titanium clip. If the distance between the clip and the ideal position was less than or equal to 1 cm, the localization procedure was completed.

After the localization procedure was completed, one-lung ventilation was restored. VATS wedge resection or segmentectomy was performed, and then the nodules were sent for intraoperative frozen section examination. Based on the results of frozen section diagnosis, VATS lobectomy was subsequently performed for patients with invasive lung cancer, or with metastasis of the 12th/13th lymph nodes. Patients with lung cancer regularly underwent systematic lymph node dissection or sampling.

### Evaluation of outcome

To evaluate the effectiveness of the localization procedure, we defined several indexes as follows:

1. Localization time: the intraoperative localization time, defined as the interval from finishing making VATS incisions to achieving an ideal position of the titanium clip.
2. Success rate of localization: defined as the number of successful localization nodules/number of all localization nodules.
3. Success rate of VATS resection: defined as the number of successful VATS resections on basis of localization/number of all successful localization nodules.

### Statistical analysis

Statistical analyses were performed using SPSS version 22.0 statistical software (IBM SPSS Statistics, Chicago, IL, USA). Continuous data are presented as mean ± standard deviation or median and range. Categorical data are presented as number (percentage, %).

## Results

### Clinical data

A total of 27 patients were included in this study, including 6 males and 21 females. The median age was 58 years (range 34–78 years). All surgeries were performed by two-port VATS, and none were converted to thoracotomy. The average operation time was 128.7 ± 46.3 min. There were no perioperative complications or deaths. The clinical data was listed in Table 1.

**Table 1:** Clinical characteristics of the patients in this study

Characteristic	Values (n = 27)
Age (years)	58 (34–78)
Gender (male/female)	6/21
Current smokers (n, %)	6 (22.2%)
Patients with COPD (n, %)	2 (7.4%)
Patients with single/with multiple nodules	19/8
Total patients/pulmonary nodules	27/35
Operation time (min)	128.7 ± 46.3
Duration of postoperative stay (day)	4 (2.6)

### Localization data

A total of 35 pulmonary nodules underwent intraoperative localization in 27 patients. The mean diameter of nodules was 10.6 ± 3.7 mm. The mean distance of the nodules to visceral pleura was 8.3 ± 8.7 mm. There were 23 (65.7%) pure GGO nodules, 8 (22.9%) mixed GGO nodules, and 4 (11.4%) solid nodules. The mean localization time was 23.3 ± 3.3 min. The median times of C-arm scanning was 3 (range 2–4) times. The median times for clipping were 2 (range 1–3) times. The success rate of localization was 100% (35/35), and the success rate of VATS resection was also 100% (35/35). No complications related to localization occurred. Final pathological results of nodules showed that 22 (62.9%) cases were invasive adenocarcinomas, 1 (2.9%) microinvasive adenocarcinoma, 5 (14.3%) adenocarcinoma in situ, 2 (5.7%) atypical adenomatous hyperplasia (AAH), 1 (2.9%) adenoma, and 4 (11.4%) inflammatory lesions. None of the patients had hilar or mediastinal lymph node metastasis. The characteristics of nodules and localization data were listed in Table 2.

**Table 2:** Characteristics of pulmonary nodules

Characteristic	Values (n = 35)
Size (mm)	10.6 ± 3.7
Distance from visceral pleura (mm)	8.3 ± 8.7
*Location of nodules *(*n*, *%*)	
Right upper lobe	15 (42.9%)
Right middle lobe	2 (5.7%)
Right lower lobe	6 (22.9%)
Left upper lobe	7 (20%)
Left lower lobe	5 (14.3%)
*Nodule density* (*n*, *%*)	
Pure GGO	23 (65.7%)
Mixed GGO	8 (22.9%)
Solid	4 (11.4%)
Localization time (min)	23.3 ± 3.3
Times of scanning of C-arm	3 (range 2–4)
Times of clipping	2 (range 1–3)
Complications (n)	0
*Pathological results* (*n*, *%*)	
Inflammatory	4 (11.4%)
Adenoma	1 (2.9%)
Atypical adenomatous hyperplasia (AAH)	2 (5.7%)
Adenocarcinoma in situ	5 (14.3%)
Microinvasive adenocarcinoma	1 (2.9%)
Invasive adenocarcinoma	22 (62.9%)

## Discussion

As small nodules were difficult to be found by touch, some VATS failed to resect the nodules completely and accurately, and some were converted to open thoracotomy [5–7]. Localization of small pulmonary nodules is quite essential and helpful. As reported before, most localization procedures were implemented from several hours to 1 or 2 days before the surgery, and the most widely used method in the clinic is preoperative CT-guided localization**.** Many materials were reported to be used in CT-guided localization procedure, including hookwire, microcoil, some liquid materials such as lipiodol, methylene blue, and so on [1–4, 8, 9]. These methods are easy to implement in most hospitals. The success rates of localization are always above 90%. CT guided localization methods need specialized interventional radiologist, and inevitably, a few of patients underwent a failure procedure for several reasons, such as dislocation, intolerance. Also, many complications may happen, such as chest pain, pneumothorax, irritant cough, hemothorax and even embolism, and it is always impossible for relocation or localization for more than one nodules. Additional, preoperative procedures were done under local anesthesia, which may lead to excessive fear and suffering for patients when they were awake. Some studies have reported intraoperative techniques to localize pulmonary nodules, which included intraoperative ultrasound and fluorescent thoracoscopic localization in a hybrid operating room [10]. Electromagnetic navigation bronchoscopy (ENB) is a real-time navigation system that uses three-dimension images generated from a preoperative chest CT scan. ENB system can create a road map through the airway anatomy to guide a catheter to the lesion of interest for intraoperative biopsy and localization. Luo K and his colleagues [11] reported 24 consecutive patients using ENB to guide a catheter adjacent to the target lesion and inject fibrin sealant mixed methylene blue for the localization of small pulmonary nodules. ENB system may reduce the risks of pneumothorax and hemothorax, which is more safe than CT-guided percutaneous puncture. However, the accuracy of localization, complicated steps and high cost limited the application of ENB in clinical.

In this study, we introduced a new simple and effective intraoperative localization for small pulmonary nodules in hybrid operating room. The procedure was undergone under thoracoscopic observation. After making the VATS ports, a titanium clip was clipped at the visceral pleura to be a marker for the nodule. VATS resection was performed next. In this study, a 100% success rate of localization and VATS resection were demonstrated. All the nodules were confirmed by pathology, and no VATS were converted to thoracotomy. There were almost no complications related to localization procedures. Therefore, this new intraoperative technique could be effectively and safely performed. The advantages of this procedure were as follows: (1) localization steps were simple, without the necessary of specialized interventional radiologist, and total operation time was not prolonged. (2) the marker was located at the visceral pleura, instead of in the pulmonary parenchyma, which could avoid the risks of pneumothorax, hemothorax and embolism. (3) the localization and resection procedure were accomplished under one time of general anesthenia, which could avoid extra suffering for patients. (4) titanium clips used in this technique were chip and accessible, and no extra expenses were needed. This localization technique was believed to have two potential disadvantages. First, this technique was not suitable for pulmonary nodules located in the inner one-third field of lung. These nodules were too far away from the visceral pleura, and markers at the visceral pleura could not indicate the nodules precisely. Second, this technique was performed in hybrid operating room, which may limit the popularity in some hospitals.

## Limitations

This study was an exploratory research in only one surgical team. Also, it was a retrospective study, with a relatively small-size sample. Therefore, selection bias was unavoidable. Prospective studies with a control group were required to fully evaluate the efficacy of this new localization technique in further research.

## Conclusions

This new intraoperative localization technique was feasible, safe and effective. All the small pulmonary nodules were localized and resected successfully. No localization related complications happened. No extra expenses were required and the intraoperative procedure could avoid extra suffering for patients.

## Data Availability

All data generated or analysed during this study are included in this published article. The datasets used during the current study are available from the corresponding author on reasonable request.
